# 2,4,6-Trimethyl­pyridinium dihydrogen phosphate

**DOI:** 10.1107/S1600536810050646

**Published:** 2010-12-18

**Authors:** Hong-Ling Cai, Jing Dai

**Affiliations:** a Ordered Matter Science Research Center, College of Chemistry and Chemical Engineering, Southeast University, Nanjing 210096, People’s Republic of China

## Abstract

In the title compound, C_8_H_9_N^+^·H_2_PO_4_^−^, both the cation and anion have crystallographically imposed mirror symmetry (all atoms apart from one O atom lie on the mirror plane). In the crystal, anions and cations are linked by O—H⋯O and π–π stacking inter­actions [centroid–centroid distance = 3.4574 (6) Å], forming chains parallel to the *b* axis. Adjacent chains are further connected by N—H⋯O hydrogen bonds into a two-dimensional network.

## Related literature

For background to the properties of pyridine salts as phase-transition dielectric materials, see: Fu *et al.* (2007 ▶, 2008 ▶, 2009 ▶); Fu & Xiong (2008 ▶). 
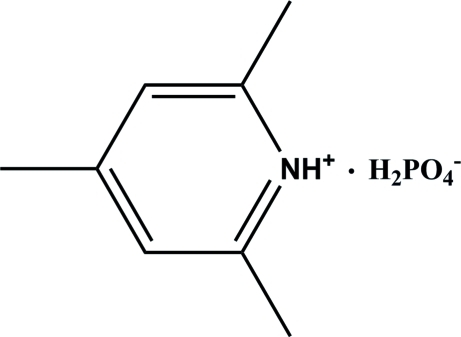


## Experimental

### 

#### Crystal data

C_8_H_9_N^+^·H_2_O_4_P^−^
*M* *_r_* = 216.15
Monoclinic, 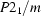

*a* = 8.6323 (17) Å
*b* = 6.7133 (13) Å
*c* = 8.6841 (17) Å
β = 100.99 (3)°
*V* = 494.02 (17) Å^3^
*Z* = 2
Mo *K*α radiation
μ = 0.27 mm^−1^
*T* = 298 K
0.30 × 0.05 × 0.05 mm

#### Data collection

Rigaku Mercury2 diffractometer
Absorption correction: multi-scan (*CrystalClear*; Rigaku, 2005 ▶) *T*_min_ = 0.910, *T*_max_ = 1.000
5154 measured reflections
1229 independent reflections
1082 reflections with *I* > 2σ(*I*)
*R*_int_ = 0.033

#### Refinement

*R*[*F* ^2^ > 2σ(*F* ^2^)] = 0.047
*wR*(*F* ^2^) = 0.144
*S* = 1.18
1229 reflections
86 parameters
1 restraint
H atoms treated by a mixture of independent and constrained refinement
Δρ_max_ = 0.45 e Å^−3^
Δρ_min_ = −0.29 e Å^−3^

### 

Data collection: *CrystalClear* (Rigaku, 2005 ▶); cell refinement: *CrystalClear*; data reduction: *CrystalClear*; program(s) used to solve structure: *SHELXS97* (Sheldrick, 2008 ▶); program(s) used to refine structure: *SHELXL97* (Sheldrick, 2008 ▶); molecular graphics: *SHELXTL* (Sheldrick, 2008 ▶); software used to prepare material for publication: *SHELXTL*.

## Supplementary Material

Crystal structure: contains datablocks I, global. DOI: 10.1107/S1600536810050646/rz2528sup1.cif

Structure factors: contains datablocks I. DOI: 10.1107/S1600536810050646/rz2528Isup2.hkl

Additional supplementary materials:  crystallographic information; 3D view; checkCIF report

## Figures and Tables

**Table 1:** Hydrogen-bond geometry (Å, °)

*D*—H⋯*A*	*D*—H	H⋯*A*	*D*⋯*A*	*D*—H⋯*A*
O2—H2⋯O3^i^	0.85 (2)	1.76 (2)	2.6054 (19)	169 (2)
N1—H1*A*⋯O1	0.86	1.75	2.602 (3)	173

Symmetry code: (i) 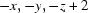.

**Fig. 1.**
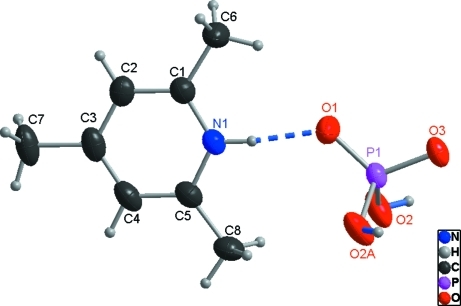
A view of the title compound with the atomic numbering scheme. Displacement ellipsoids were drawn at the 30% probability level. Symmetry code: (A) x, 0.5-y, z.

**Fig. 2.**
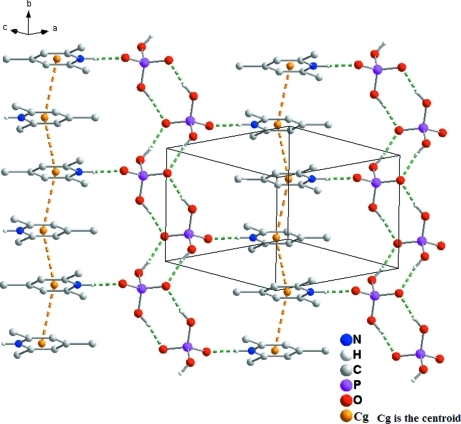
The crystal packing of the title compound, showing the two-dimensional network. H atoms not involved in hydrogen bonding (dashed line) have been omitted for clarity.

## supplementary crystallographic information

### Comment

Salts of pyridine have attracted attention as phase transition dielectric materials for their applications in memory storage (Fu *et al.* 2007; Fu & Xiong 2008; Fu *et al.* 2008; Fu *et al.* 2009). With the purpose of obtaining new phase transition crystals of 2,4,6-trimethylpyridine salts, their interaction with various acids has been studied and we have elaborated a series of new materials with this organic molecule. In this study, we describe the crystal structure of the title compound, 2,4,6-trimethylpyridinium dihydrogen phosphate.

The asymmetric unit is composed of half an H_2_PO_4_^-^ anion and half a C_8_H_9_N^+^ cation (Fig. 1), both anion and cation being located on a mirror plane. The geometric parameters are in the normal range. In the crystal structure, the anions are linked into chains parallel to the *b* axis by O—H···O hydrogen bonds (Table 1). The cations also are connected into chains along the *b* axis by π–π stacking interactions with centroid-to-centroid distances of 3.4574 (6) Å. The cationic and anionic chains further interact through N—H···O hydrogen bonds (Fig. 2), forming a two-dimensional network.

### Experimental

The commercial 2,4,6-trimethylpyridine (3 mmol) was dissolved in water/H_3_PO_4_ (50:1 *v*/*v*) solution. The solvent was slowly evaporated in air affording colourless block-shaped crystals of the title compound suitable for X-ray analysis.

The dielectric constant of title compound as a function of temperature indicates that the permittivity is basically temperature-independent, suggesting that this compound should be not a real ferroelectrics or there may be no distinct phase transition occurred within the measured temperature range. Similarly, below the melting point (413 K) of the compound, the dielectric constant as a function of temperature also goes smoothly, and there is no dielectric anomaly observed (dielectric constant equaling to 6.6 to 8.9).

### Refinement

All H atoms attached to C and N atoms were fixed geometrically and treated as riding with C–H = 0.93–0.96 Å, N–H = 0.86 Å, and with *U*_iso_(H) = 1.2 *U*_eq_(C, N) or 1.5 *U*_eq_(C) for methyl H atoms. The H atom of the H_2_PO_4_^-^ anion was located in difference Fourier maps and freely refined, with the O—H distance constrained to 0.86 Å..

### Crystal data

**Table:**

C_8_H_9_N^+^·H_2_O_4_P^−^	*F*(000) = 226
*M**_r_* = 216.15	*D*_x_ = 1.453 Mg m^−^^3^
Monoclinic, *P*2_1_/*m*	Mo *K*α radiation, λ = 0.71073 Å
Hall symbol: -P 2yb	Cell parameters from 1229 reflections
*a* = 8.6323 (17) Å	θ = 3.1–27.5°
*b* = 6.7133 (13) Å	µ = 0.27 mm^−^^1^
*c* = 8.6841 (17) Å	*T* = 298 K
β = 100.99 (3)°	Block, colorless
*V* = 494.02 (17) Å^3^	0.30 × 0.05 × 0.05 mm
*Z* = 2	

### Data collection

**Table:**

Rigaku Mercury2 diffractometer	1229 independent reflections
Radiation source: fine-focus sealed tube	1082 reflections with *I* > 2σ(*I*)
graphite	*R*_int_ = 0.033
Detector resolution: 13.6612 pixels mm^-1^	θ_max_ = 27.5°, θ_min_ = 3.1°
CCD profile fitting scans	*h* = −11→11
Absorption correction: multi-scan (*CrystalClear*; Rigaku, 2005)	*k* = −8→8
*T*_min_ = 0.910, *T*_max_ = 1.000	*l* = −11→11
5154 measured reflections	

### Refinement

**Table:**

Refinement on *F*^2^	Primary atom site location: structure-invariant direct methods
Least-squares matrix: full	Secondary atom site location: difference Fourier map
*R*[*F*^2^ > 2σ(*F*^2^)] = 0.047	Hydrogen site location: inferred from neighbouring sites
*wR*(*F*^2^) = 0.144	H atoms treated by a mixture of independent and constrained refinement
*S* = 1.18	*w* = 1/[σ^2^(*F*_o_^2^) + (0.0788*P*)^2^ + 0.1875*P*] where *P* = (*F*_o_^2^ + 2*F*_c_^2^)/3
1229 reflections	(Δ/σ)_max_ < 0.001
86 parameters	Δρ_max_ = 0.45 e Å^−^^3^
1 restraint	Δρ_min_ = −0.29 e Å^−^^3^

### Special details

**Table:**

Geometry. All e.s.d.'s (except the e.s.d. in the dihedral angle between two l.s. planes) are estimated using the full covariance matrix. The cell e.s.d.'s are taken into account individually in the estimation of e.s.d.'s in distances, angles and torsion angles; correlations between e.s.d.'s in cell parameters are only used when they are defined by crystal symmetry. An approximate (isotropic) treatment of cell e.s.d.'s is used for estimating e.s.d.'s involving l.s. planes.
Refinement. Refinement of *F*^2^ against ALL reflections. The weighted *R*-factor *wR* and goodness of fit *S* are based on *F*^2^, conventional *R*-factors *R* are based on *F*, with *F* set to zero for negative *F*^2^. The threshold expression of *F*^2^ > σ(*F*^2^) is used only for calculating *R*-factors(gt) *etc*. and is not relevant to the choice of reflections for refinement. *R*-factors based on *F*^2^ are statistically about twice as large as those based on *F*, and *R*- factors based on ALL data will be even larger.

### Fractional atomic coordinates and isotropic or equivalent isotropic displacement parameters (Å^2^)

**Table:**

	*x*	*y*	*z*	*U*_iso_*/*U*_eq_	Occ. (<1)
N1	0.3386 (3)	0.2500	0.5841 (3)	0.0298 (5)	
H1A	0.2650	0.2500	0.6385	0.036*	
C3	0.5712 (4)	0.2500	0.4112 (4)	0.0382 (7)	
C2	0.4135 (4)	0.2500	0.3392 (4)	0.0378 (7)	
H2A	0.3862	0.2500	0.2303	0.045*	
C1	0.2974 (3)	0.2500	0.4270 (3)	0.0333 (6)	
C5	0.4890 (3)	0.2500	0.6600 (4)	0.0338 (6)	
C4	0.6066 (3)	0.2500	0.5731 (4)	0.0383 (7)	
H4A	0.7115	0.2500	0.6242	0.046*	
C7	0.6980 (5)	0.2500	0.3145 (5)	0.0548 (10)	
H7A	0.7986	0.2500	0.3845	0.082*	
H7B	0.6891	0.1332	0.2496	0.082*	0.50
C8	0.5179 (4)	0.2500	0.8351 (4)	0.0456 (8)	
H8A	0.6296	0.2500	0.8750	0.068*	
H8B	0.4717	0.3668	0.8715	0.068*	0.50
C6	0.1257 (4)	0.2500	0.3578 (4)	0.0505 (9)	
H6A	0.0675	0.2500	0.4417	0.076*	
H6B	0.0992	0.3668	0.2945	0.076*	0.50
P1	0.09606 (8)	0.2500	0.89656 (8)	0.0280 (3)	
O1	0.0994 (3)	0.2500	0.7269 (3)	0.0515 (7)	
O2	0.19545 (18)	0.0720 (3)	0.9782 (2)	0.0494 (5)	
O3	−0.0654 (2)	0.2500	0.9371 (2)	0.0343 (5)	
H2	0.145 (3)	−0.024 (3)	1.010 (3)	0.066 (9)*	

### Atomic displacement parameters (Å^2^)

**Table:**

	*U*^11^	*U*^22^	*U*^33^	*U*^12^	*U*^13^	*U*^23^
N1	0.0261 (11)	0.0273 (11)	0.0368 (12)	0.000	0.0079 (9)	0.000
C3	0.0383 (16)	0.0230 (13)	0.0587 (19)	0.000	0.0226 (14)	0.000
C2	0.0431 (17)	0.0325 (15)	0.0408 (16)	0.000	0.0156 (13)	0.000
C1	0.0325 (14)	0.0303 (14)	0.0375 (15)	0.000	0.0078 (12)	0.000
C5	0.0289 (14)	0.0270 (13)	0.0446 (16)	0.000	0.0044 (12)	0.000
C4	0.0256 (13)	0.0288 (14)	0.0609 (19)	0.000	0.0091 (13)	0.000
C7	0.0469 (19)	0.0458 (19)	0.082 (3)	0.000	0.0385 (19)	0.000
C8	0.0344 (16)	0.056 (2)	0.0419 (17)	0.000	−0.0032 (13)	0.000
C6	0.0344 (16)	0.080 (3)	0.0366 (16)	0.000	0.0040 (13)	0.000
P1	0.0243 (4)	0.0276 (4)	0.0340 (4)	0.000	0.0102 (3)	0.000
O1	0.0383 (12)	0.0835 (19)	0.0353 (12)	0.000	0.0131 (9)	0.000
O2	0.0297 (8)	0.0390 (9)	0.0826 (13)	0.0050 (7)	0.0181 (8)	0.0211 (8)
O3	0.0267 (10)	0.0300 (10)	0.0493 (12)	0.000	0.0152 (9)	0.000

### Geometric parameters (Å, °)

**Table:**

N1—C5	1.339 (4)	C7—H7A	0.9601
N1—C1	1.343 (4)	C7—H7B	0.9600
N1—H1A	0.8600	C8—H8A	0.9600
C3—C4	1.381 (5)	C8—H8B	0.9600
C3—C2	1.385 (5)	C6—H6A	0.9600
C3—C7	1.501 (4)	C6—H6B	0.9600
C2—C1	1.371 (4)	P1—O1	1.479 (2)
C2—H2A	0.9300	P1—O3	1.502 (2)
C1—C6	1.489 (4)	P1—O2^i^	1.5603 (17)
C5—C4	1.376 (4)	P1—O2	1.5603 (16)
C5—C8	1.493 (4)	O2—H2	0.85 (2)
C4—H4A	0.9300		
			
C5—N1—C1	123.0 (3)	C3—C4—H4A	119.5
C5—N1—H1A	118.5	C3—C7—H7A	108.3
C1—N1—H1A	118.5	C3—C7—H7B	110.1
C4—C3—C2	117.9 (3)	H7A—C7—H7B	109.5
C4—C3—C7	121.7 (3)	C5—C8—H8A	109.2
C2—C3—C7	120.4 (3)	C5—C8—H8B	109.6
C1—C2—C3	120.6 (3)	H8A—C8—H8B	109.5
C1—C2—H2A	119.7	C1—C6—H6A	108.5
C3—C2—H2A	119.7	C1—C6—H6B	109.9
N1—C1—C2	119.0 (3)	H6A—C6—H6B	109.5
N1—C1—C6	117.4 (3)	O1—P1—O3	115.41 (13)
C2—C1—C6	123.5 (3)	O1—P1—O2^i^	109.81 (9)
N1—C5—C4	118.5 (3)	O3—P1—O2^i^	110.37 (8)
N1—C5—C8	117.4 (3)	O1—P1—O2	109.81 (9)
C4—C5—C8	124.1 (3)	O3—P1—O2	110.37 (8)
C5—C4—C3	121.1 (3)	O2^i^—P1—O2	99.97 (14)
C5—C4—H4A	119.5	P1—O2—H2	117 (2)

Symmetry codes: (i) *x*, −*y*+1/2, *z*.

### Hydrogen-bond geometry (Å, °)

**Table:**

*D*—H···*A*	*D*—H	H···*A*	*D*···*A*	*D*—H···*A*
O2—H2···O3^ii^	0.85 (2)	1.76 (2)	2.6054 (19)	169 (2)
N1—H1A···O1	0.86	1.75	2.602 (3)	173.

Symmetry codes: (ii) −*x*, −*y*, −*z*+2.
